# Ancient Human Migrations to and through Jammu Kashmir- India were not of Males Exclusively

**DOI:** 10.1038/s41598-017-18893-8

**Published:** 2018-01-16

**Authors:** Indu Sharma, Varun Sharma, Akbar Khan, Parvinder Kumar, Ekta Rai, Rameshwar N. K. Bamezai, Miguel Vilar, Swarkar Sharma

**Affiliations:** 1 Human Genetics Research Group, School of Biotechnology, Shri Mata Vaishno Devi University, Katra, 182320 India; 2 Department of Zoology, University of Jammu, Jammu and Kashmir, 180006 India; 3 Institute of Human Genetics, University of Jammu, Jammu and Kashmir, 180006 India; 4 School of Life Sciences, Jawaharlal Nehru University, New Delhi, 110067 India; 5 The Genographic Project, National Geographic Society, Washington, DC, 20036 USA

## Abstract

Jammu and Kashmir (J&K), the Northern most State of India, has been under-represented or altogether absent in most of the phylogenetic studies carried out in literature, despite its strategic location in the Himalayan region. Nonetheless, this region may have acted as a corridor to various migrations to and from mainland India, Eurasia or northeast Asia. The belief goes that most of the migrations post-late-Pleistocene were mainly male dominated, primarily associated with population invasions, where female migration may thus have been limited. To evaluate female-centered migration patterns in the region, we sequenced 83 complete mitochondrial genomes of unrelated individuals belonging to different ethnic groups from the state. We observed a high diversity in the studied maternal lineages, identifying 19 new maternal sub-haplogroups (HGs). High maternal diversity and our phylogenetic analyses suggest that the migrations post-Pleistocene were not strictly paternal, as described in the literature. These preliminary observations highlight the need to carry out an extensive study of the endogamous populations of the region to unravel many facts and find links in the peopling of India.

## Introduction

India has served as a major corridor for modern human migration and was amongst the first regions of the world populated by humans once they left Africa^1,2^. It is a land with extensive human diversity^3^ which has witnessed many prehistoric and historic migrations and invasions across the ages due to its geographical location and natural bounty. The migrations and invasions have resulted in the high cultural and social diversity of the region^4^. A favored hypothesis in literature is that migrations were not uniform; men were migratory and women were sedentary^5^ as the invasions were mostly male dominated. Also, it is believed that after the initial Pleistocene settlement, the migrants from central and west Asia were primarily males^6,7^ and the initial female population in India may have been small in numbers^8,9^ and thus limited maternal gene flow in and out of India^10^. It is popularly concluded that maternal gene pool of the Indian subcontinent is relatively ancient and conserved as compared to the paternal genetic component^3^.

Jammu and Kashmir (J&K) is the Northern most region of India, located on the crossroads of Eurasia, bound by China and Tibet from North-East and Afghanistan and Pakistan on the North-West. The state is divided into three sub-regions (Jammu, Kashmir and Ladakh), which are geographically isolated. This region has been suggested to have served as a corridor for various migrations and immigrations in the mainland India and Eurasia^11^. The population groups speak languages mainly belonging to the Indo-European linguistic family. Another language group in the region, the Tibeto-Burman, is predominantly spoken in the Leh-Ladakh region whereas, some population groups from the Kashmir region speak various dialects belonging to a distinct Indo-Aryan language group known as ‘Dardic’.

It has been observed that inhabitant populations resided in small pockets and remained isolated for centuries due to various social practices. Many historical migrations/invasions impacted the social structure of the valley. One of the documented invasions followed by settling down has been that of Greek Emperor Alexander the Great, who invaded North India and his army stayed in the region for more than three years, and some of them building Greek cities in what is modern day Afghanistan and the Punjab area^12^. In addition, the Northern and Southern routes from China fostered missionary activities that would reach India during which Kashmir served as a gateway to the subcontinent. Today, there are indelible imprints of these East Asian on the socio-cultural ethos of the Kashmir valley, like the burial of dogs and perforated stone knives, both characteristics of the North Neolithic Chinese culture^13^. Archaeological evidence of early settlement and migration is seen in Mesolithic stone tools and handmade pottery from the Burzahom site in Kashmir which indicate that the early occupants were hunter-gatherers. The pottery may be indicative of food storage and thus signs of early farming, as well. These recovered stone tools and pottery from the site are in close affinity with that of Swat Valley in Pakistan^13^. The excavation of axes/bowls made of bronze from Karakoram (now in Pakistan but a part of J&K before, 1947) suggest Bronze Age presence and likely western influence in J&K^14^. Further, several branches of the legendary Silk Road network connected J&K with rest of Central Asia, through which people traveled for trade and pilgrimage^15^. Interestingly, the question remains how did these historical events of acculturation impact the gene pool of the region? Several studies have been carried out targeting Indian populations^16–18^, however, the region of J&K has always been mostly excluded or restricted by limited sampling, thus remaining most under studied. We emphasize that this region must be targeted as a whole, to understand the genetic context of Indian populations and its connection to the greater Eurasian continent.

## Result and Discussion

In this study, in order to address the compelling question as to whether, the maternal gene pool of J&K was conserved across millennia, as expected from other phylogenetic studies of Indian populations, we targeted the mitogenomes, the strongest genetic tool for tracing direct maternal inheritance^19^, of 83 individuals belonging to different ethnic groups across Jammu and Kashmir.

**Figure 1:**
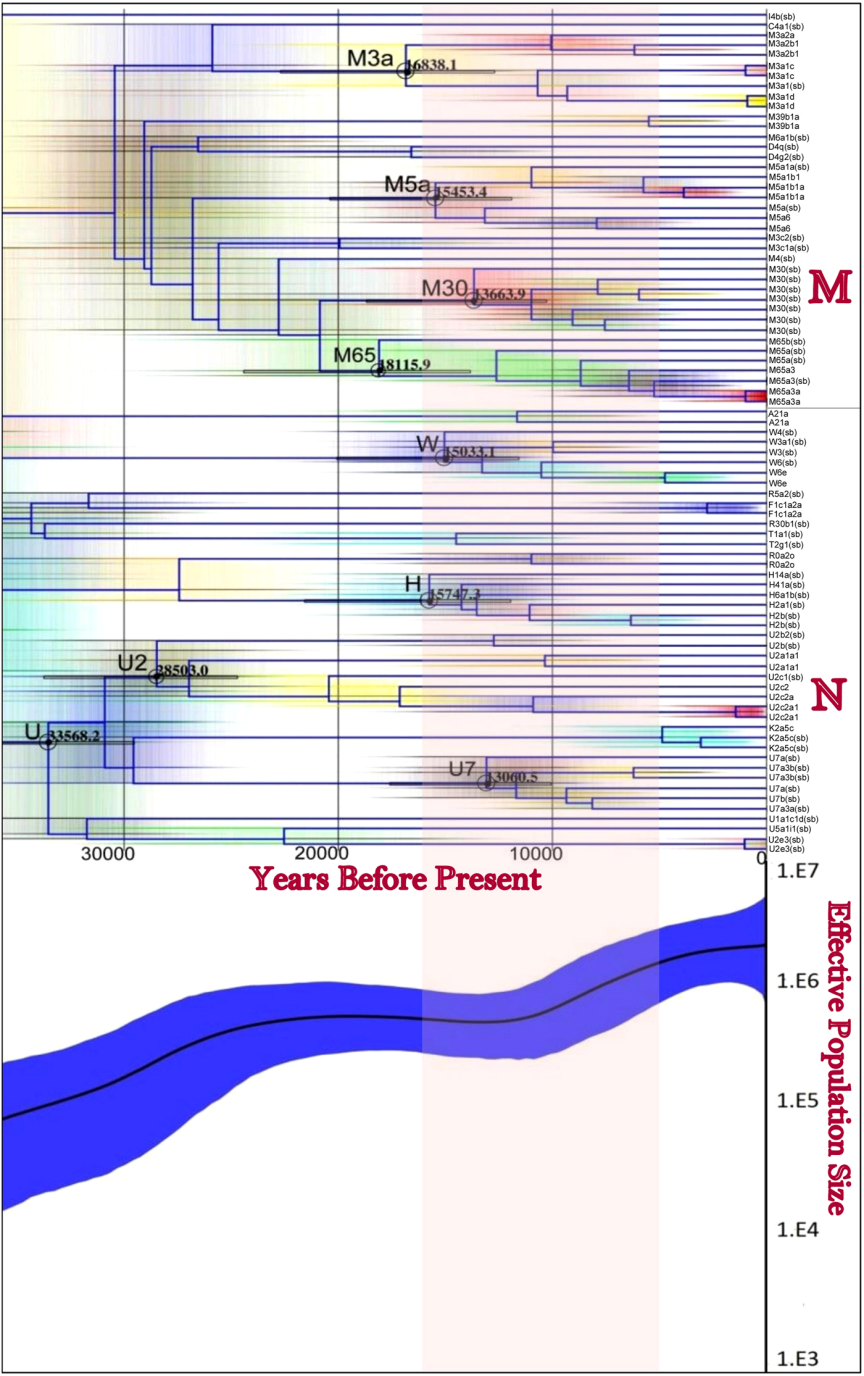
Phylogenetic tree and Bayesian Skyline plot based on complete mitogenomes of Jammu and Kashmir. The branching patterns of phylotree and spike in BSP shows expansion of maternal gene pool of Jammu and Kashmir past Last Glacial Maximum and early Holocene around a time period of 15,000–8000 YBP (highlighted). The solid line in the middle in BSP is the median estimate, while outer thin lines show the 95% highest posterior density (HPD) limits. Good convergence was achieved by applying the HKY^72^ and strict clock models^79^, With the prior mutation rates having a mean of (2.67 × 10^−8^)^73^.

From the data set, we identified a high diversity of maternal HGs in the region as the 83 mitogenomes included types from haplogroup (HG) to M, U, H, W, R, K, F, D, T, A, C and I. These major HG, included the lineages from as far as both Europe and Central Asia. Further, the phylogenetic diversity documented here has not been observed before in Indian populations. From the 83 mitogenomes, we identified 19 novel lineages, belonging to the following groups: M3a1c, M3a1d, M3a2b, M3a2b1, M65a3, M65a3a, M5a1b1, M5a1b1a, M5a6, M39b1a, W6e, F1c1a2a, U2a1a1, U2c2, U2c2a, U2c2a1, K2a5c, A21a and R0a2o. HGs were assigned to each individual according to the nomenclature of PhyloTree.org^20^ (mtDNA tree Build 17). These novel lineages were only designated when at least 2 individuals could be found belonging to that lineage (Table 1). Interestingly, some of the lineages called for a rearrangement of the existing phylogenetic tree (mtDNA tree build 17) to accommodate the previously unknown diversity. To avoid confusion, we named the lineages to follow the existing nomenclature of phylotree (build 17) but highlighted the branches that need rearrangement in the manuscript. In addition, a few individuals were observed with distinct haplotypes than those in existing phylotree (mtDNA tree build 17) and in literature. However, as these set of variants were observed only once, and thus were considered as possible private polymorphisms, and were added as new branches in the existing maternal phylogenetic tree **(**supplementary Data 1 and 2). It is possible that these branches are potentially new haplogroups with deep (Pleistocene) time differentiation (Fig. 1), yet we lacked replicate samples and further evidence to be characterized as such, due to a smaller sample size in the present preliminary study from the region.

**Table 1:** Novel HGs with common variants in the samples. Haplotypes shared by at least two unrelated individual were considered to constitute ‘new haplogroups’.

S. No.	Haplogroup	Common Variants Characterized for Novel Haplogroup	ID of Individuals Sharing Common Variants
**1**.	M3a1c	T152C, A9051G, A9218G	NG11, NG17
**2**.	M3a1d	A7670C, T14208C, A15649G, T16234C	NG65, NG105
**3**.	M3a2b	C7967T	NG48, NG51, NG137
**4**.	M3a2b1	T15458C	NG51, NG137
**5**.	M65a3*	G9254A	NG50, NG100, NG197
**6**.	M65a3a^#^	T15479C	NG50, NG100
**7**.	M5a1b1	C3954T, T9833C	NG107, NG79, NG42
**8**.	M5a1b1a	A15902G	NG107, NG79
**9**.	M5a6	T4500C, G10589A, C11203T	NG66, NG69
**10**.	M39b1a	T158A, A1446T, G3531A, T16304C	NG98, NG129
**11**.	W6e	G143A	NG2, NG25
**12**.	F1c1a2a	A234G,	NG63, NG49
**13**.	U2a1a1	T15629C	NG29, NG64
**14**.	U2c2	C13934T	NG4, NG47, NG178, NG115
**15**.	U2c2a	T4772C, C16188T, A16207G	NG4, NG47, NG178
**16**.	U2c2a1	T63C, C64T, G9554A, A15954G, G16213A	NG4, NG178
**17**.	K2a5c	A15799G	NG76, NG97, AR18
**18**.	A21a	C12603T, T16092C	NG40, NG143
**19**.	R0a2o	T11152C	NG193, NG211

^*^To be renamed as M65a1b, ^#^ to be renamed as M65a1b1, in revised phylotree.

### Distribution of Indian specific haplogroups

Previous literature has reported that 60% of the maternal lineages are from the ancient macrohaplogroup M in the Indian subcontinent^21^. Further, some of the sub-HGs of M are *in-situ* and deep rooted in India^22^. These basal branches of macrohaplogroup M, which are old and highly diverse, suggest the initial settlement of India was likely during the earliest waves of modern human from Africa^23^. We found novel lineages of HG M in the J&K region in high frequency and diversity. For example: two new sub-HGs of M3a1 and M3a2 found in South India^23^ were observed as Sub-HGs, M3a1c and M3a1d, defined by variant motif T152C, A9051G, A9218G and A7670C, T14208C, A15649G, T16234C, respectively along with one additional sub-branch. Similarly, M3a2 can now be classified into two HGs: M3a2b and M3a2b1, defined by variant C7967T and T15458C, respectively, with one sub-branch. One sub-branch was added to each HG: M3c1a, M3c2, M4 and M65b. We also found novel sub-haplogroups in M65a, M65a3 further differentiating into M65a3a, and with additional three sub-branches in M65a1, as per existing tree (mtDNA tree build 17). However introduction of these branches call for rearrangement of the tree as: M65a2 to M65a1a; M65a3 to M65a1b; M65a3a to M65a1b1 (Supplementary Data 1). Incidentally, HG M65 is believed to have arisen in the area of the Indian subcontinent during the Upper Paleolithic period. However, it is reported to be present in people inhabiting present day Tibet, Himalayas, India and Pakistan^24^. Five sub-branches were added to M30 and one to M30b in the present study. One sub-branch was added to M5a1a; whereas, M5a1b that is reported in South India^25^, was also found in J&K with two sub-HGs: M5a1b1 and M5a1b1a, defined by variants C3954T, T9833C and A15902G, respectively. Two sub-branches were also added to M5a6 and one to M5a and a new sub-haplogroup, M5a6a, was added to M5a6, defined by variants T4500C and G10589A. M39 has been documented in the tribal populations of India^23^. We found sub-branch of M39b1, classified to M39b1a and defined by variants T58A, A1446T, G3531A and T16304C. Despite the fact that the frequency of HG M is higher in the South than North India^10,26^, we have observed diverse and distinct sub-HGs of HG M in this preliminary study (Supplementary Data 1), indicating a deep in time differentiation and migration to the region. These observations highlight the possibility that a lot has to be discovered in the region, which may have great implications in understanding the phylogeny of HG M in India.

Also seen in previous work from India, we found HG U to be predominant and the second most frequent and highly diverse group in the present study. HG U is known to be distributed geographically from Europe and North Africa through India and Central Asia^21,27,28^. It is the second most frequent maternal lineage in India as it is also in Europe^29^. The autochthonous HGs like U2a, U2b and U2c were observed in Indian populations^28,30,31^. To these HGs, newer sub HGs are added in the present study. U2a, reported to be present in Pakistan^32^, showed a new sub-haplogroup, designated as U2a1a and defined by variant T15629C. Two sub-branches were added to the HG U2b2 and one sub-branch to U2c1. Basal HGs U2c2, U2c2a, U2c2a1 were also observed and defined by variants: C13934T, and T4772C, C16188T, A16207G, and T63C, C64T, G9554T, A15954G, G16213A, respectively. A sub-branch was added to U2e1′2′3 and U7a3. Further, two sub-branches were added to U7a, U7a3b; and one sub-branch each to U7a3a, U7b, U5a1i1 and U1a1c1d (Supplementary Data 2). HG U7 has been found in various parts of India, Pakistan and Iran^10^ but is also widely distributed across Europe, near East and South Asia^33^. Additionally, one sub-branch was added to each HG R5a2 and R30b1; both R5 and R30 are common Indian HGs^31^.

### Presence of non-Indian haplogroups in J&K

We identified various other HGs (sub-branches and some new HGs) of R, W, H, I, T, K, C, A, F and D but with relatively less frequency. These HGs appeared in low frequency individually, which in the present study may be due to a small sample size. However, all these groups taken together represented 29/83 studied samples (34.9%), thus more than a third of lineages were contributed from outside of India. Some of these HGs were mainly the West Eurasian and North-East Asian HGs, rarely have been found in India. Moreover, given the extent of diversity, these are some of the most interesting observations made in the present study. Various phylogenetic analyses (phylogenetic tree and Bayesian analysis), indicate an introduction of this genetic component during post Last Glacial Maximum (<14000 YBP) to early Holocene period (>8000 YBP) (Fig. 1), which may coincide with dispersal of language and adoption of agriculture^34^. Thus, the diversity observed could have arisen as a result of large scale female migration to the region in post-settlement times, but could also be differentiation of many of the lineages in the region, *in-situ*, since Pleistocene settlement. In order to make this differentiation, we need further and more extensive study of the region, including possible ancient DNA contributions.

In the present study, various new HGs belonging to macro haplogroup N were observed. A new sub-haplogroup was added to R0a2: the R0a2o, defined by variant T11152C and two sub-branches (Supplementary Data 2). R0 is a West Eurasian haplogroup^35^, and has also been reported in population of Iran^36^. Further, one sub-branch each was added to HGs, W4 and W6, and two sub-braches to W3. Additionally, a new sub-haplogroup, not reported earlier, was defined as W6e with basal variant G143A and further differentiation in the region with variants G7521A, T16189C, C16355T and T16362C (Supplementary data 2). The W haplogroup has been reported to be the one of the dominant groups in Iran, Pakistan^10^; and is also widely distributed with low frequencies over European continent, the Near East and West Asia^37^. Similarly haplogroup H, commonly present in Caucasus and widely spread within Europe with a rare presence in India^38^, was observed in the present study. Further, one sub-branch was added to each HG H2a and H6a1b: and two sub-branches to each sub-haplogropu H2b and H41a. Also, one sub-branch was added to each, I4b and T2g1 (I4b and T2 are present in Europe, the Near East and the Caucasia^39,40^). Another novel sub-haplogroup of K2a5: K2a5c, defined by A15799G at basal position with further differentiation possessing variants C9356T and A3397G C7241T was reported for the first time in India. HG K2a5 has predominantly been documented in West Eurasians^41^. European gene pool has been affected by major gene flow from steppe region of North of the Caucasus and has resulted in a shift in the genetic profile of Europeans during Bronze Age^42,43^.

It has also been suggested that groups from the steppe regions of Eurasia were the source population of Indo-European languages to Europe^42^ and also likely to South Asia^44^. This linguistic spread could explain the presence of these European lineage in South Asia. The other plausible migration of maternal European gene pool to this region could have been from the Southern coast of Iran, Afghanistan; and present Pakistan through Baluchistan, acting as conduits for human dispersals^36,45^. These geographically adjacent regions were connected with the State of Jammu and Kashmir and were also regions that saw similar historical gene flow from Europe as India did. therefore, the extent of diversity, we propose, may either indicate differentiation of various population groups in the neighboring region^36^ or be the remnants of natural diversity associated with various migrations of high magnitude through various invaders, like Alexander the Great, the Persian Empire, Arabs, Turks, Syrians and Afghans. Thus, the historical migrants too could have introduced the genetic component of West and Central Asia to this region^12^ which interestingly was not exclusively that of males.

In addition, many Eastern Asian haplogroups were also observed. Ancient branches of HG C(C4a1) present in Eastern Asia, may have been introduced to J&K with movement south from central and Eastern Asia with the concurrent expansions in Northern Asia^46^. HG A one of the major haplogroup in Tibetans^47^ and a common haplogroup in Northern and Eastern Asia^48^, was found in the form of one new sub-haplogroup, A21a, defined by C12603T and T16092C variants. The two HGs A and C have also been reported in Tibet with a high frequency among Sherpa population^47^. Likewise, HG F1c, which is predominantly present in the Tibetans^49^ and Chinese populations^50^ was observed in J&K in sub-branches as F1c1a2a, defined by variants A234G and G1927A. Within haplogroup D4 seen in Han Chinese populations^51^, we added sub-branches to HG D4g2a1c and D4q. The presence of these lineages representing North East Asian gene pool in J&K may have been contributed through the Silk Route when people migrated in and out of the region.

### Affinity with the Global Populations

In order to visualize the maternal closeness of J&K population with various population of the world, genetic distances amongst different populations were estimated as Fst values^52^. In addition to J&K samples from present study samples from population sets, Africa(AFR)^53^, Caucasian(CAU)^54^, China(CH)^50^, Europe(EUR)^53^, India(IN1)^31^, India(IN2)^55^, Japan(JAP)^56^, assorted Jew(JEW)^57^, Onge(ONG)^58^, Pakistan(PAK)^32^, Papua New Guinea(PNG)^59^ and Native Americans(AME)^53^ were included for the analysis. Fst values were further used to generate Multi Dimensional Scaling (MDS) plot (Supplementary Fig. 1). First plot was generated by taking population of JK as whole. It was observed that JK population set clusters in between Asian and European population sets (Supplementary Fig. 1a). To have a better understanding of genetic affinity of samples bearing known Indian and non-Indian haplogroups found in J&K population, samples were divided into two sets JK1 and JK2. JK1 set constituted the HGs known in literature majorly as Indian population specific haplogroups (M, U2 & U7). JK2 set was comprised of all other HGs (A, C, D, F, H, I, K, R, T, U1, U5, W), found in the study. Interestingly, JK2 clustered with European and Caucasian population set (Supplementary Fig. 1b).

### Maternal population expansion in the region

The initial settlement of humans in South Asia is reported to have occurred between 40,000–70,000 years before present (YBP)^60^, whereas the initial maternal colonization in Indian subcontinent is reported to have occurred around 40,000–45,000 YBP^61^. We found various lineages of macrohaplogroup M and N, clearly indicating high diversity and maternal gene flow to and from the region as discussed and also indicated by large negative values of Fu’s Fs statistics and the highly significant values of Tajima’s D (Supplementary Table 1). This substantiated the pattern of a maternal population expansion in Jammu and Kashmir population. Bayesian Skyline Analysis (BSA) and phylogenetic tree branching patterns indicated a gradual population growth over 35,000 YBP but an expansion episode can be detected around 15,000–8000 YBP, which could be attributed to an expansion after the Last Glacial Maximum (Fig. 1) that could be *insitu* differentiation or associated with agriculture and language dispersal also indicated by some of the archeological evidences from the region.

### Coalescence Age estimates of major Haplogroups

We tried to estimate the coalescent ages (expressed as years before present, YBP) of major haplogroups found in our sample set using BEAST software^62^. The most frequent haplogroups of macrohaplogroup M in the present study were M3a, M5, M30, and M65; whereas, of macrohaplogroup N were U, U2, U7, W and H (Supplementary Table 2). Most of these coalescence time periods in the present study were in agreement with the estimates reported in literature. The coalescence time period of M3a has been reported as 16400 YBP^22^, and observed as 16838.1 YBP with 95% HPD of (12654.3–22671.5). Similarly, M5a has been estimated with an age of 23100 YBP^22^, whereas we observed 15453.4 YBP with 95% HPD of (11878.9–20377.2). In case of M30, reported to have originated 15400 YBP^22^, showed in our estimates its age as 13663.9 YBP with 95% HPD of (10237.1–18628.2), in the region. The coalescence age of M65 which has been reported as 20600 YBP^34^, in our study has been observed as 18115.9 YBP with 95% HPD of (13786.7–24394.0).

One of the major haplogroups, haplogroup U and some of its lineages which have been reported to be differentiated in the Indian subcontinent^21,63,64^, showed its coalescence age of 33568.2 YBP with 95% HPD of (29494.3–39721.8) in J&K. The mean age of HG U has been reported as 46531.1^41^ and the coalescence age of U2 has been reported as 42805.7^41^ YBP. The age for the latter when calculated in the region was 28503.0 YBP with 95% HPD of (24666.1–33724.1). Further, HG U7 which has been known to be differentiated in south Asia with the age of 15600 YBP^33^, showed the coalescence age estimate of 13060.5 with 95% HPD of (10014.2–17556.7), in the present study. In contrast, the absolute coalescence age estimates of European haplogroups in J&K were not consistent with those in Europe, though 95% HPD values overlapped; West Eurasian haplogroup W has been reported in Europe with the age of 18400 YBP^37^, we observed an age of 15033.1 YBP with 95%HPD of (11539.9–20022.5). While as, Haplogroup H which has been estimated to originate around (12846.0) YBP^41^, but our estimates show it around 15747.3 YBP in J&K with 95% HPD of (11917.5–21518.1). Thus, the diversity and deep in time coalescence ages of known European HGs in the region highlight the importance of the region for exploring its plausibility as differentiation ground of many maternal lineages that might have migrated to Europe from the region post glacial maximum. Overall, this age estimation has provided an overview of the expansion of mtDNA haplogroups in the region, indicating population expansion in the J&K region at different time intervals, most likely with post-settlement gene flow east from western Eurasia to India.

## Conclusion

To conclude, the extent of presence of variants defining novel HGs or personal variants indicate high diversity in maternal genetic component of the population of J&K. Statistical analyses indicate that maternal population in J&K have undergone expansion, along with other regions of Indian sub-continent^9^. However, signatures of maternal gene pool expansion in the region past LGM and early Holocene era are also seen, and this is a unique observation for the present study. These distinct signatures and maternal lineages, never reported before in India, apparently suggest that this region might have served as a corridor, yet also as a reservoir for many unreported lineages.

The overall diversity seen in the maternal gene pool of J&K suggests that the migrations to and through this region were not exclusively of males. This data has refined the existing phylogenetic tree and added to the information further diversity of mtDNA in Indian populations. Further, this preliminary study highlights the importance of the region and emphasizes that the populations of this region should be studied extensively to understand the gene pool of Indian populations. Along with the Y chromosomal and mtDNA markers, a study of autosomal markers is also warranted in these population groups. It is anticipated to help in finding some of the missing links in the evolution of modern humans and their migratory history to and from the mainland India and the Indian subcontinent, a future perspective of our study. Further, we would like to emphasize that the endogamous populations should be studied with respect to their individual evolutionary and migration histories, rather than pooling these together as one group, an underlying drawback that has plagued many of the Indian population based studies in the past, diluting individual signatures and masking stories their DNA has to tell.

## Materials and Methods

### Sample collection

Samples were collected with informed consent from all participants. The study was approved by Institutional Ethical Review Board (IERB) of Shri Mata Vaishno Devi University. All experimental protocols were conducted according to the guidelines and regulations set by the IERB. The samples represent cumulatively the ethnic population of J&K, but belong to various endogamous groups (or subgroups), such as, Brukpa, Bakkarwal, Brahmin, Gujjar, Kashmiri Pandit, Kashmiri Muslim, Khatri, Lohar, Rajput, Sikh and SC (subgroups- Bhagat, Balmiki, Charmark, Mahasha, Screra, Tradiye) from three provinces of J&K i.e. Jammu, Kashmir and Ladakh.

### Complete mtDNA sequencing

Complete mitochondrial sequencing was done by targeted amplification of the mitochondrial genome by long-range PCR, from genomic DNA. The mitochondrial genome was amplified as two long overlapping fragments of 9Kb each. Equimolar concentrations of the two fragments were pooled and taken for library preparation using Illumina TruSeq DNA sample preparation kits. The LR PCR products were fragmented to 300–400 bp size in Covaris M220 followed by end repair, adenylation and Illumina adaptor ligation. The adaptor ligated libraries were further amplified and size selected using Ampure XP beads. The libraries were then sequenced to more than 1000 × coverage on Illumina sequencing platform (HiSeq. 2500). The sequences obtained were aligned to revised Cambridge mitochondrial reference genome (RCRS) using BWA program^65,66^ and analyzed using Picard and GATK-Lite toolkit^67,68^, the variant called were crosschecked with RSRS^20^.

Relevant variants were annotated using published variants in literature and MitoMap database^20^. The homozygous variants with read depth more than 100 were visually confirmed using IGV 2.3^69^ to be considered as variants for analyses (Supplementary Data 3). Haplogroup were assigned to particular sequence using (https://dna.jameslick.com/mthap/)^70^ based on phylotree 17^20^. The haplogroup/sub-haplogroup frequency is given in Supplementary Table 3. We estimated various statistical values to elucidate the extent of diversity. Fu’s Fs statistics and Tajimas’s D values were calculated by DNASP v5^71^.

### Phylogenetic analysis of Mitogenomes

Bayesian Skyline Analysis (BSA) was also done, to calculate effective population size with time using BEAST v1.8.2 suite^62^ (Bayesian Evolutionary Analysis Sampling Trees) software. We performed several analyses on the partitioned mtDNA (partition of mtDNA was done using a custom python script into control region, tRNA plus rRNA regions, first, second, and third positions of codons in the protein coding regions)^33^, with a strict molecular clock and uncorrelated lognormal relaxed clock with HKY (Hasegawa Kishino and Yano) model^72^ of nucleotide substitutions. The reasonable ESS (Estimated Sample Size) values i.e (>200) were obtained with strict clock. Bayesian Skyline algorithm was selected in BEAST to generate Bayesian Skyline Plot. Each MCMC (Markov Chain Monte Carlo) sample was based on a run of 30000000 generations sampled every 3000 steps, with the first 3000000 generations regarded as burn-in. Runs were made with a mutation rate (2.67 × 10^−8^)^73^ and (2.74 × 10^−8^)^74^, however results presented here are based on (2.67 × 10^−8^)^73^. We visualized BSP with Tracer software v1.6 (Supplementary Fig. 2). Phylogenetic analysis of J&K Mitogenomes was also done using Densitree^75^. The age of most recent common ancestor (TMRCA) and 95% highest posterior density intervals of major haplogroups were calculated using human mitochondrial evolutionary rates 2.67 × 10^−8^ with European mitogenomes^73^ (Supplementary Fig. 3). Fst values were used to generate Multi Dimensional Scaling (MDS plot) using SPSS statistics software v.20^76^. Complete mtDNA sequences for various population groups were downloaded from (http://www.mtdb.igp.uu.se/)^77^ and aligned using DNasp v.5^71^. The Fst values and statistical significance was estimated by permutation analysis, using 10,000 permutations by Arlequin software v.3.5^78^.

### Accession codes at GenBank

KX467262, KX467263, KX467264, KX467265, KX467266, KX467267, KX467268, KX467269, KX467270, KX467273, KX467274, KX467275, KX467276, KX467277, KX467278, KX467279, KX467280, KX467281, KX467282 KX467283, KX467284, KX467285, KX467286, KX467287, KX467288, KX467289, KX467290, KX467291, KX467292, KX467293, KX467294, KX467290, KX467291, KX467292, KX467293, KX467294, KX467295, KX467296, KX467297, KX467298, KX467299, KX467300, KX467301, KX467302, KX467303, KX467304, KX467305, KX467306, KX467307, KX467308, KX467309, KX467310, KX467311, KX467312, KX467313, KX467314, KX467315, KX467316, KX467317, KX467318, KX467319, KX467320, KX467321, KX467322, KX467323, KX467324, KX467325, KX467326, KU178917, KU178918, KU178919, KU178920, KU178921, KU178922, KU178923, KU178924, KU178925, KU178926, KU178927, KU178928, KU178929, KU178930, KU178931.

## Electronic supplementary material

Supplementary File

Supplementary Data 1.

Supplementary Data 2.

Supplementary Data 3.
