# Supplementary material for: Ancient Human Migrations to and through Jammu Kashmir- India were not of Males Exclusively
Source: Sci Rep. 2018 Jan 16;8:851. doi: 10.1038/s41598-017-18893-8 (PMC5770440; doi:10.1038/s41598-017-18893-8)
Supplement: Supplementary file 1 — Supplementary File [file 41598_2017_18893_MOESM1_ESM.doc]

**Ancient Human Migrations to and through Jammu Kashmir- India were not of Males Exclusively.**

Indu Sharma^1+^, Varun Sharma^1+^, Akbar Khan^2^, Parvinder Kumar^2, 3^, Ekta Rai^1^, Rameshwar NK Bamezai^4^, Miguel Vilar^5^, Swarkar Sharma^1*^

+equal contribution

Affiliations:

1. Human Genetics Research Group, Department of Biotechnology, Shri Mata Vaishno Devi

University, Katra, 182320, India.

1. Department of Zoology, University of Jammu, Jammu and Kashmir, 180006, India.
2. Institute of Human Genetics, University of Jammu, Jammu and Kashmir, 180006, India.
3. School of Life Sciences, Jawaharlal Nehru University, New Delhi, 110067, India.
4. The Genographic Project, National Geographic Society, USA.

*Corresponding Author:

**Dr. Swarkar Sharma**

Coordinator, Human Genetics Research Group

Department of Biotechnology, Shri Mata Vaishno Devi University, Katra, J&K, India

Mobile: +91-9419955636 Ph: +91-1991-285535//285525 Ext. 2385

Email: [swarkar.sharma@smvdu.ac.in](mailto:Swarkar.sharma@smvdu.ac.in)

a

b

**Supplementary Figure 1. MDS plot of Jammu and Kashmir with other population sets**. For a comparative analysis Mutli Dimensional Scaling was done. Plot was generated from the Fst values (provided in Supplementary Table 4a and 4b) representing complete mtDNA sequences from different population groups retrieved from mtDNA database[^1^](#_ENREF_1) and 83 mitogenomes from Jammu and Kashmir (details in material and method section). (a) Plot was generated using 13 different population sets and Jammu and Kashmir as a whole set. (b) Plot of Jammu and Kashmir as two sets: JK1 and JK2. Samples belonging to reported mtDNA haplogroups as Indian specific i.e HGs M, U2 and U7 were included as group JK1. All other samples were included as group JK2. The purpose was to evaluate the closeness of JK2 set specifically with worldwide representative populations.


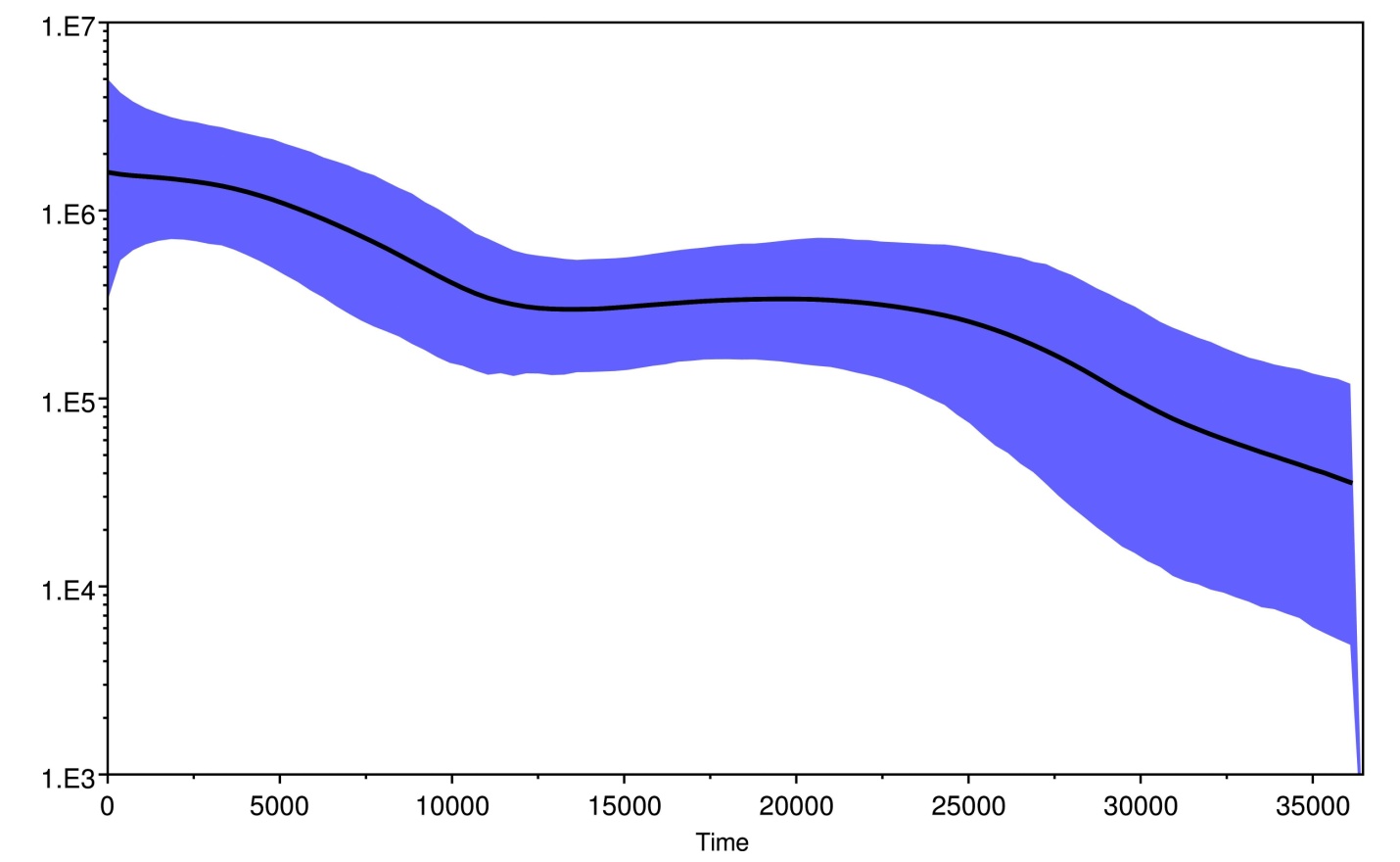
**Supplementary Figure 2. Bayesian Skyline plot of population of Jammu and Kashmir.** Plot was generated to see Effective population size (Ne) with time and shows a population expansion around a time period of 35000 YBP. The plot was generated using BEAST[^2^](#_ENREF_2) software. mtDNA sequences were partitioned into five parts: control region, tRNA plus rRNA regions, first, second and third positions of codons in the protein coding regions using a custom designed python script. Various analyses were done using a mutation rates having a mean of (Fu et al.)[**^3^**](#_ENREF_3) i.e 2.67×10^-^**^8^** good convergence was achieved by applying the HKY[^4^](#_ENREF_4) and strict clock model[^5^](#_ENREF_5). The plot was visualized in Tracer software v1.6. The same has been incorporated along with mtDNA phylogenetic tree in Figure 1 for better understanding of population expansion.

Ne


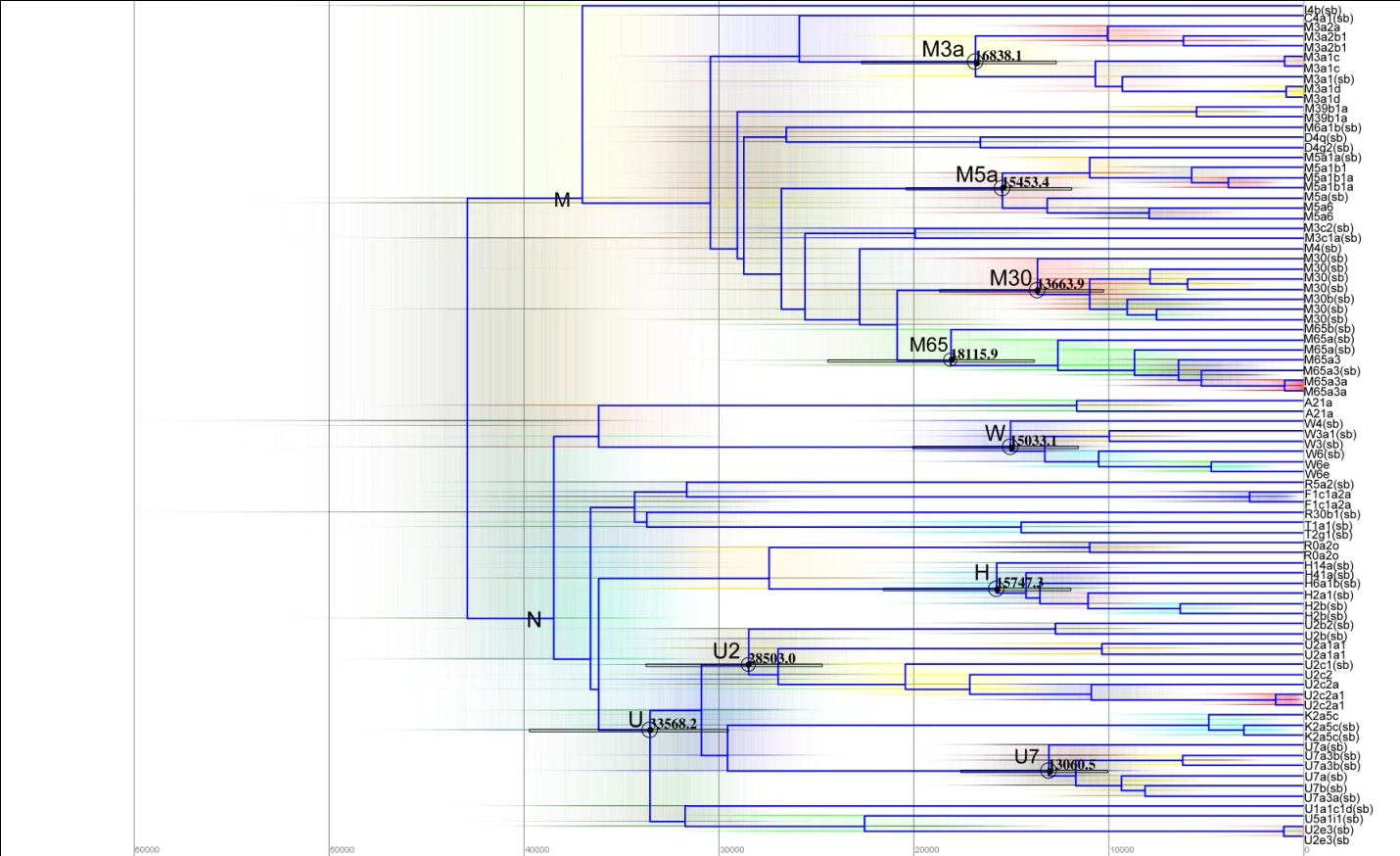


**Supplementary Figure 3. mtDNA phylogeny of Jammu and Kashmir population.** Phylogenetic tree of 83 Jammu and Kashmir Mitogenomes was generated using densitree[^6^](#_ENREF_6), the major haplogroups of M and N are shown with a circle. The age of most recent common ancestor (TMRCA) and 95% highest posterior density intervals shown were calculated using mitochondrial evolutionary calibrated in[^3^](#_ENREF_3). The (sb) in the labels designate Sub-branch. The same phylogenetic tree has been provided in Figure 1 along with BSP (supplementary Figure 2), highlighting diversity and population expansion events.

**Supplementary Table1. Statistics based on complete mtDNA of Jammu and Kashmir Mitogenomes.** The software DNASP v5 was used to identify the number of polymorphic sites and number of mutations, to calculate nucleotide diversity, Tajima’s D values, Fu’s Fs statistics**.**

| Number of sequences  (Na) | Number polymorphic site(s) (N_b_) | Total no. of mutations  (Nc) | Average no of nucleotide difference | Nucleotide diversity (π) | Tajima's D (D) | Fu's Fs statistics (Fs) | P value |
| --- | --- | --- | --- | --- | --- | --- | --- |
| 83 | 543 | 552 | 36.02057 | 0.00218 | -2.32974 | -43.738 | <0.05 |

**Supplementary Table2.**

TMRCA of major clades of J&K population calculated using human mitochondrial evolutionary rates calibrated in (Fu et al.)[^3^](#_ENREF_3) with 95% HPD intervals.

| Major clade of J&K population | Age calculated in years |
| --- | --- |
| M3a | 16838.1 (12654.3 - 22671.5) |
| M5a | 15453.4 (11878.9 - 20377.2) |
| M30 | 13663.9 (10237-1 - 18628.2) |
| M65 | 18115.9 (13786.7 - 24394.0) |
| W | 15033.1 (11539.9 - 20022.5) |
| H | 15747.3 (11917.5 - 21518.1) |
| U | 33568.2 (29494.3 - 39721.8) |
| U2 | 28503.0 (24666.1 - 33724.1) |
| U7 | 13060.5 (10014.2 - 17556.7) |

**Supplementary Table3.**

The number and frequency of HGs found in the present study.

| Haplogroups | Number of samples | Frequency (%) found in our data |
| --- | --- | --- |
| M | 35 | 42.18 |
| U | 19 | 22.9 |
| H | 6 | 7.24 |
| W | 6 | 7.24 |
| R | 4 | 4.82 |
| K | 3 | 3.62 |
| F | 2 | 2.4 |
| D | 2 | 2.4 |
| T | 2 | 2.4 |
| A | 2 | 2.4 |
| C | 1 | 1.2 |
| I | 1 | 1.2 |
| Total | 83 |  |

| Populations | Afr | JK | Cau | Pak | Png | Jap | Jew | Ch | Ong | Eur | Ame | IN1 | IN2 |
| --- | --- | --- | --- | --- | --- | --- | --- | --- | --- | --- | --- | --- | --- |
| Afr | 0 |  |  |  |  |  |  |  |  |  |  |  |  |
| JK | 0.55311 | 0 |  |  |  |  |  |  |  |  |  |  |  |
| Cau | 0.48192 | 0.13659 | 0 |  |  |  |  |  |  |  |  |  |  |
| Pak | 0.4613 | 0.19106 | 0.21205 | 0 |  |  |  |  |  |  |  |  |  |
| Png | 0.42319 | 0.19014 | 0.2337 | 0.27855 | 0 |  |  |  |  |  |  |  |  |
| Jap | 0.47123 | 0.19112 | 0.35416 | 0.39869 | 0.20572 | 0 |  |  |  |  |  |  |  |
| Jew | 0.53528 | 0.30958 | 0.38329 | 0.30506 | 0.37993 | 0.48854 | 0 |  |  |  |  |  |  |
| Ch | 0.4024 | 0.1066 | 0.15398 | 0.21294 | 0.0551 | 0.14239 | 0.33199 | 0 |  |  |  |  |  |
| Onge | 0.43788 | 0.19264 | 0.43419 | 0.46327 | 0.23516 | 0.19976 | 0.55619 | 0.1914 | 0 |  |  |  |  |
| Eur | 0.47266 | 0.12032 | -0.00634 | 0.12501 | 0.2063 | 0.32743 | 0.23551 | 0.12024 | 0.39211 | 0 |  |  |  |
| Ame | 0.44444 | 0.15917 | 0.27225 | 0.32646 | 0.16802 | 0.04722 | 0.42761 | 0.0724 | 0.20841 | 0.24276 | 0 |  |  |
| IN1 | 0.51435 | 0.15037 | 0.01681 | 0.2918 | 0.26909 | 0.40405 | 0.45482 | 0.18624 | 0.51745 | 0.05359 | 0.31566 | 0 |  |
| IN2 | 0.48477 | 0.14611 | 0.39537 | 0.44886 | 0.22787 | 0.14235 | 0.53086 | 0.16896 | 0.16667 | 0.36657 | 0.16454 | 0.45034 | 0 |

**Supplementary Table 4a.** Fst values estimated by permutation analysis, using 10,000 permutations by Arlequin software v.3.5[^7^](#_ENREF_7) representing complete mtDNA sequences from 13 different population groups retrieved from mtDNA database[^1^](#_ENREF_1) and 83 mitogenomes from Jammu and Kashmir as a whole set. The Supplementary Figure 1a is based on the values given below.

Africa(AFR)[^8^](#_ENREF_8), Jammu and Kashmir (JK), Caucasian(CAU)[^9^](#_ENREF_9), China(CH)[^10^](#_ENREF_10), Europe(EUR)[^8^](#_ENREF_8), India(IN1)[^11^](#_ENREF_11), India(IN2)[^12^](#_ENREF_12), Japan(JAP)[^13^](#_ENREF_13), assorted Jew(JEW)[^14^](#_ENREF_14), Onge(ONG)[^15^](#_ENREF_15), Pakistan(PAK)[^16^](#_ENREF_16), Papua New Guinea(PNG)[^17^](#_ENREF_17) and Native Americans(AME)[^8^](#_ENREF_8)

**Supplementary Table 4b** Fst values estimated by permutation analysis, using 10,000 permutations by Arlequin software v.3.5[^7^](#_ENREF_7) representing complete mtDNA sequences from 13 different population groups retrieved from mtDNA database[^1^](#_ENREF_1) and 83 mitogenomes from Jammu and Kashmir as two sets: JK1 and JK2.

| Populations | Afr | Cau | Ch | Eur | IN1 | IN2 | Jap | Jew | Ong | Pak | Png | Ame | JK1 | JK2 |
| --- | --- | --- | --- | --- | --- | --- | --- | --- | --- | --- | --- | --- | --- | --- |
| Afr | 0 |  |  |  |  |  |  |  |  |  |  |  |  |  |
| Cau | 0.48192 | 0 |  |  |  |  |  |  |  |  |  |  |  |  |
| Ch | 0.4024 | 0.15398 | 0 |  |  |  |  |  |  |  |  |  |  |  |
| Eur | 0.47266 | -0.00634 | 0.12024 | 0 |  |  |  |  |  |  |  |  |  |  |
| IN1 | 0.51435 | 0.01681 | 0.18624 | 0.05359 | 0 |  |  |  |  |  |  |  |  |  |
| IN2 | 0.48477 | 0.39537 | 0.16896 | 0.36657 | 0.45034 | 0 |  |  |  |  |  |  |  |  |
| Jap | 0.47123 | 0.35416 | 0.14239 | 0.32743 | 0.40405 | 0.14235 | 0 |  |  |  |  |  |  |  |
| Jew | 0.53528 | 0.38329 | 0.33199 | 0.23551 | 0.45482 | 0.53086 | 0.48854 | 0 |  |  |  |  |  |  |
| Ong | 0.43788 | 0.43419 | 0.1914 | 0.39211 | 0.51745 | 0.16667 | 0.19976 | 0.55619 | 0 |  |  |  |  |  |
| Pak | 0.4613 | 0.21205 | 0.21294 | 0.12501 | 0.2918 | 0.44886 | 0.39869 | 0.30506 | 0.46327 | 0 |  |  |  |  |
| Png | 0.42319 | 0.2337 | 0.0551 | 0.2063 | 0.26909 | 0.22787 | 0.20572 | 0.37993 | 0.23516 | 0.27855 | 0 |  |  |  |
| Ame | 0.44444 | 0.27225 | 0.0724 | 0.24276 | 0.31566 | 0.16454 | 0.04722 | 0.42761 | 0.20841 | 0.32646 | 0.16802 | 0 |  |  |
| JK1 | 0.5721 | 0.24948 | 0.1488 | 0.22633 | 0.26794 | 0.11554 | 0.19008 | 0.39093 | 0.18688 | 0.27107 | 0.22721 | 0.17832 | 0 |  |
| JK2 | 0.51701 | 0.06686 | 0.12773 | 0.05845 | 0.08547 | 0.27101 | 0.26348 | 0.28834 | 0.29129 | 0.17681 | 0.21025 | 0.20577 | 0.14507 | 0 |

Africa(AFR)[^8^](#_ENREF_8), Jammu and Kashmir (JK1 and JK2), Caucasian(CAU)[^9^](#_ENREF_9), China(CH)[^10^](#_ENREF_10), Europe(EUR)[^8^](#_ENREF_8), India(IN1)[^11^](#_ENREF_11), India(IN2)[^12^](#_ENREF_12), Japan(JAP)[^13^](#_ENREF_13), assorted Jew(JEW)[^14^](#_ENREF_14), Onge(ONG)[^15^](#_ENREF_15), Pakistan(PAK)[^16^](#_ENREF_16), Papua New Guinea(PNG)[^17^](#_ENREF_17) and Native Americans(AME)[^8^](#_ENREF_8)

References
